# Digital Visual Assessment of Tooth Wear: Practical Comparison of BEWE, Simplified TWI and TWES 2.0 on Intraoral Scans with Exploratory Sex-Stratified Observations

**DOI:** 10.3390/dj14050264

**Published:** 2026-05-02

**Authors:** Maria Lorens, Iwona Tomaszewska

**Affiliations:** 1 Tomaszewska Stomatologia, al.Juliusza Słowackiego 58/4, 30-004 Cracow, Poland; 2 Department of Medical Education, Centre of Innovative Medical Education, Jagiellonian University Medical College, Medyczna Street 7, 30-688 Cracow, Poland; im.tomaszewska@uj.edu.pl

**Keywords:** Basic Erosive Wear Examination (BEWE), intraoral scans, sex differences, simplified Tooth Wear Index (sTWI), tooth wear, Tooth Wear Evaluation System 2.0 (TWES 2.0)

## Abstract

**Objectives:** This study aimed to evaluate and compare the performance of three tooth wear indices—BEWE, simplified TWI (sTWI), and TWES 2.0—based on the visual assessment of digital intraoral scans. A secondary exploratory objective was to examine unadjusted sex-stratified score distributions. **Methods:** This retrospective study included 246 anonymized intraoral scans obtained using Medit i700 and iTero Element 2 systems. All scans were independently evaluated by two calibrated examiners using the BEWE, simplified TWI (sTWI), and TWES 2.0 indices. Scoring was performed through visual assessment of the digital models, without applying automated measurements or software-assisted analysis tools. Unadjusted sex-stratified patterns were explored using mixed-effects linear models, with a significance threshold set at *p* < 0.05. Estimated marginal means were calculated, and graphical approaches, including heatmaps, were used to present score distributions and inter-examiner variability. **Results:** The indices exhibited different scoring characteristics. BEWE produced the most consistent sextant-based patterns, with low variability between examiners. TWES 2.0 showed generally stable scoring, although with slightly greater dispersion, particularly in posterior sextants. In contrast, sTWI demonstrated the highest variability and more pronounced surface-specific differences, especially on occlusal and palatal surfaces. Sex-stratified analyses indicated minor and inconsistent variations. Although isolated statistically significant findings were observed (BEWE Sextant 6; TWES 2.0 Sextant 5), they did not form a consistent or interpretable pattern. **Conclusions**: Assessment based on digital intraoral scans highlighted differences in scoring behavior depending on the index used. BEWE demonstrated the most stable scoring patterns, TWES 2.0 showed moderate consistency, while sTWI was associated with the greatest variability. Differences observed in sex-stratified analyses were minor and lacked consistency. Clinical significance: BEWE enables consistent scoring when applied to digital scans and may be appropriate for monitoring purposes. TWES 2.0 provides a structured approach to screening. The higher variability observed with sTWI indicates that caution is warranted when it is used exclusively on digital models. Overall, the choice of index has a substantial impact on the assessment of tooth wear in digital workflows.

## 1. Introduction

Tooth wear is increasingly regarded as a common clinical and epidemiological concern, affecting approximately 30–50% of adults, although reported prevalence varies according to population characteristics, diagnostic criteria, and the scoring system applied [1,2]. An increase has also been noted in younger age groups, highlighting the importance of early identification, standardized follow-up, and preventive strategies [3,4]. Tooth wear is a chronic and irreversible loss of enamel and dentin resulting from erosion, attrition, and abrasion, which frequently occur together, and it may lead to hypersensitivity, impaired function, and complex restorative demands [5,6,7].

Although tooth wear has been widely investigated, its consistent assessment remains challenging because of its multifactorial nature and marked inter-individual variation. Dietary acids, reflux, parafunctional habits, oral hygiene practices, and demographic factors have all been linked to tooth wear, but findings remain inconsistent across studies [4,8,9]. Some reports indicate that males may present with greater wear than females; however, these associations are often influenced by age, masticatory forces, and lifestyle-related variables [2,8,10]. Since retrospective anonymized datasets frequently do not contain these covariates, sex-related differences identified in digital studies should be interpreted as exploratory and unadjusted [9].

The expansion of digital dentistry has created new opportunities for more standardized assessment [1,11,12]. Intraoral scanners produce detailed three-dimensional models that enable retrospective scoring, magnified inspection, repeated evaluation, and application of multiple indices without requiring additional patient appointments [13,14]. Digital datasets may therefore improve reproducibility and expand possibilities for epidemiological research.

Among the indices available for assessing tooth wear, the Basic Erosive Wear Examination (BEWE), simplified Tooth Wear Index (sTWI), and Tooth Wear Evaluation System 2.0 (TWES 2.0) are commonly used in both clinical and research settings [15,16,17,18,19,20]. The 2017 European Consensus Statement recognized these indices as key tools for evaluating the severity and distribution of tooth wear [21,22]. Nevertheless, they differ in design and intended use: BEWE serves as a rapid risk-oriented screening tool, sTWI enables site-specific surface recording, and TWES 2.0 provides a structured modular framework. Their comparability within a fully digital workflow has not yet been sufficiently explored, and the available evidence on scan-based application remains limited and heterogeneous [15,23,24,25,26].

Accordingly, the primary aim of the present study was to compare the performance of BEWE, sTWI, and TWES 2.0 when used on intraoral scans. A secondary exploratory aim was to investigate unadjusted sex-stratified score patterns within the constraints of an anonymized dataset.

## 2. Materials and Methods

### 2.1. Study Design and Ethical Approval

Ethical approval was obtained from the Bioethics Committee of the District Medical Chamber in Kraków, Poland (approval No. L.dz.OIL/KBL/22/2025, 27 May 2025).

This retrospective observational study analyzed anonymized intraoral scans to compare the performance of three tooth wear indices—BEWE, sTWI, and TWES 2.0—when applied to digital models. The study followed the principles of the Declaration of Helsinki. Because the dataset consisted of anonymized retrospective records, individual patient consent was not required; permission to use the scans was provided by all participating dental clinics.

A total of 246 intraoral scans, each corresponding to one adult patient (≥18 years), were included in the study. Only one scan per patient was included, and duplicate records or repeated scans from the same individual were excluded. Scans were acquired using Medit i700 scanners (Medit Corp., Seoul, Republic of Korea) in two clinics and iTero Element 2 scanners (Align Technology Inc., San Jose, CA, USA) in one clinic. Scanner type was not treated as an analytical variable, as both systems provide high-resolution full-arch meshes suitable for tooth wear scoring.

Sex information was available for 195 patients: 129 females (66.2%) and 66 males (33.8%). For 51 patients (20.7%), sex was unreported or unavailable in the anonymized dataset and therefore coded as “unknown” (Table 1). Sex was used only for the exploratory stratification of tooth wear scores. Age and other clinical cofactors (e.g., bruxism, diet, occlusion, systemic conditions) were not available for anonymized scans; consequently, all sex-related analyses are descriptive and unadjusted.

Only scans from adult patients aged 18 years or older were included. Eligible scans were required to display at least 14 teeth and to provide a minimum of 50% visibility of enamel and dentin surfaces to allow reliable assessment. Full-arch or near–full-arch scans were accepted, provided that sufficient anatomical detail was present for sextant-level scoring [11,27]. In this study, near–full-arch scans were defined as scans covering the majority of the dental arch, with only minimal missing regions, provided that all sextants remained assessable. Scans were excluded if extensive restorations obscured the tooth surfaces, if one or more sextants were missing or incompletely captured, or if major scanning artefacts prevented accurate visualization of morphological features.

All scans were exported in high-resolution mesh format (.stl or .ply) [12] and analyzed using Media Link software (version 3.3.6; Medit Corp., Seoul, Republic of Korea). To standardize visualization conditions, all evaluations were performed using the same type of monitor and resolution under controlled ambient lighting. Examiners were allowed unrestricted rotation, zooming, and panning of the 3D models. No slicing tools, measurement aids, or automated analysis functions were used. All scores were based exclusively on visual inspection of the digital models, without the use of automated, quantitative, or software-assisted measurement methods.

### 2.2. Examiner Training and Calibration

Two trained examiners with more than five years of clinical experience in restorative and preventive dentistry independently evaluated all scans using BEWE, sTWI, and TWES 2.0 (screening module). Before formal data collection, both examiners participated in a calibration session using a set of representative intraoral scans not included in the final dataset. During calibration, the scoring criteria for each index were reviewed, and borderline cases were discussed until consensus was reached [16,20,28,29,30]. Calibration was repeated until satisfactory consensus was reached. After this phase, all study scans were scored independently; examiners were blinded to each other’s scores and did not have access to previously recorded measurements.

Formal inter-rater agreement metrics (e.g., Cohen’s kappa or Kendall’s coefficient) were not calculated, as the primary aim of the study was to compare index performance rather than examiner reliability.

### 2.3. Definition and Diagnostic Indices Used in This Study

Tooth wear was defined according to the FDI World Dental Federation (2024) as the non-carious, irreversible loss of dental hard tissues resulting from mechanical and/or chemical processes, including abrasion, attrition, and erosion [31]. The 2017 European Consensus Statement emphasized BEWE, TWI, and TWES as key tools for clinical assessment. TWES has since been updated to TWES 2.0, which was used in the present study [15,21]. Because intraoral scan–derived digital meshes do not allow reliable differentiation between underlying etiological mechanisms, each index in this study was applied strictly on the basis of visible morphological surface loss, following the original scoring criteria specified for BEWE, sTWI, and TWES 2.0 [15,23,24].

#### 2.3.1. BEWE (Basic Erosive Wear Examination)

The Basic Erosive Wear Examination (BEWE) is a sextant-based screening tool used for the rapid assessment of erosive tooth wear [16,17,32]. The dentition is divided into six sextants (excluding third molars), and in each sextant the surface exhibiting the greatest degree of wear is recorded. The sextants correspond to: maxillary right posterior (14–17), maxillary anterior (13–23), maxillary left posterior (24–27), mandibular left posterior (34–37), mandibular anterior (33–43), and mandibular right posterior (44–47) [16,23,24,32].

Scoring is performed using a four-level ordinal scale reflecting increasing severity of surface loss:

0—no visible wear;
1—initial loss of surface texture;
2—distinct hard tissue loss affecting < 50% of the surface;
3—hard tissue loss affecting > 50% of the surface.

Although BEWE scores are commonly summed to generate a cumulative value for risk assessment, this approach was not applied in the present study. Instead, sextant scores were analyzed individually to allow direct comparison with sTWI and TWES 2.0, both of which provide site-specific outcomes. This strategy preserved scoring resolution and enabled consistent comparison across indices.

#### 2.3.2. Simplified Tooth Wear Index (sTWI)

The Tooth Wear Index (TWI), proposed by Smith and Knight in 1984, is a widely used system for assessing tooth wear associated with abrasion, attrition, and erosion [18,33,34]. Because full-mouth application of TWI is detailed and time-intensive, simplified adaptations have been introduced for screening and epidemiological purposes. In the present study, a simplified version (sTWI) was applied to maxillary and mandibular anterior teeth (canines and incisors; 13–23, 33–43) as well as first molars.

The applied sTWI protocol was based on previously described simplified approaches used in epidemiological studies, focusing on canines, central incisors, and first molars to balance feasibility with sensitivity to early and moderate dentin exposure [28,30,33].

Each selected surface was evaluated using a five-point ordinal scale consistent with the original TWI definitions:

0—no visible wear;
1—wear limited to enamel;
2—dentin exposure affecting up to one-third of the surface;
3—dentin exposure affecting more than one-third but less than two-thirds of the surface;
4—severe wear with dentin exposure exceeding two-thirds of the surface or substantial loss of anatomical contour.

Surfaces that could not be reliably assessed due to extensive restorations, defects, or artifacts were coded as “R” and excluded from further analysis. In line with the approach used for BEWE in this study, sTWI scores were not aggregated across teeth; instead, each surface was treated as an independent observation. This allowed direct comparison between indices while preserving spatial resolution for statistical evaluation.

The simplified protocol was selected due to its suitability for retrospective intraoral scan analysis and its practical applicability in digital datasets. By limiting the number of evaluated teeth, sTWI enables efficient scoring while maintaining clinically relevant sensitivity to early and moderate wear, making it appropriate for comparison with BEWE and the TWES 2.0 screening module [28,30].

#### 2.3.3. TWES 2.0 (Screening Module)

The Tooth Wear Evaluation System (TWES) was first described by Wetselaar and Lobbezoo in 2016 as a structured and modular framework for the assessment of non-carious tooth wear [20,35,36]. In 2020, the system was updated to TWES 2.0, with refinements in severity grading, clearer diagnostic categories, and improved clinical applicability [29]. TWES 2.0 comprises two components: the Screening Module, intended for rapid initial assessment, and the Status Module, designed for more detailed diagnosis and treatment planning [15,20,36,37,38,39,40,41].

In the present study, only the Screening Module was used. This approach reflects both the intended function of the module and the limitations associated with digital scan–based research. The Screening Module is suitable for intraoral scans because it relies exclusively on visible morphological features. In contrast, the Status Module requires clinical information that is not available in anonymized STL/PLY datasets, including symptom assessment, etiological factors, tactile evaluation, and differentiation of restorative materials.

As in the BEWE protocol, the dentition was divided into six sextants, excluding third molars. The sextants were defined as follows: maxillary right posterior (14–17), maxillary anterior (13–23), maxillary left posterior (24–27), mandibular left posterior (34–37), mandibular anterior (33–43), and mandibular right posterior (44–47). In accordance with TWES 2.0 recommendations, an additional score was recorded separately for the palatal surfaces of the maxillary anterior sextant, as this region is particularly prone to erosive and mixed-type wear.

Within each sextant, the surface showing the highest degree of wear was recorded using the TWES 2.0 five-point ordinal scale. The system distinguishes between occlusal/incisal and non-occlusal surfaces:

Occlusal/incisal surfaces:
0—no visible wear;
1—wear limited to enamel;
2—dentin exposure with ≤1/3 loss of crown height;
3—crown height loss > 1/3 and <2/3;
4—crown height loss ≥ 2/3.
Non-occlusal (buccal/lingual/palatal) surfaces:
0—no visible wear;
1—wear limited to enamel;
2—dentin exposure < 50% of the surface;
3—dentin exposure ≥ 50% of the surface;
4—complete enamel loss or pulp exposure.

In line with TWES methodology, sextant scores were analyzed independently, without summation. Each sextant was treated as a separate observational unit for statistical analysis.

The Status Module was not included, as its application requires full clinical context, including symptom evaluation, etiological assessment, and detailed surface interpretation, which cannot be reliably obtained from digital meshes lacking color, translucency, and tactile information. Previous studies have demonstrated that while TWES 2.0 performs reliably in clinical and cast-based settings, only the Screening Module can be consistently applied to intraoral scan–derived models [20].

Therefore, restricting the analysis to the Screening Module ensured methodological consistency, reproducibility between examiners, and alignment with the study objective of comparing tooth-wear indices in a digital workflow. 

### 2.4. Scoring Structure and Data Handling

For each participant, BEWE yielded six sextant-level scores, sTWI generated multiple tooth- and surface-specific scores, and TWES 2.0 produced six sextant scores with an additional palatal-anterior score for the maxillary sextant. Missing values were documented when a sextant or tooth surface could not be assessed due to incomplete scanning, artifacts, or extensive restorative coverage. Only valid observations were retained for index-specific analyses to ensure consistency across datasets.

All scans were evaluated independently by both examiners under blinded conditions. The examiners had no access to patient characteristics, their own prior measurements, or each other’s scores. The presentation order of scans was randomized separately for each examiner to minimize sequence effects. Once recorded, scores were not modified or re-evaluated.

The scoring criteria were applied strictly according to the original definitions of each index, as described in the cited references.

### 2.5. Statistical Analysis 

Statistical analyses were performed using R software (version 4.4). Descriptive statistics were calculated for all indices, including the number of observations, median, interquartile range, mean, standard deviation, and minimum–maximum values. Score distributions and potential skewness were visualized using boxplots to provide an overview of central tendency and variability.

Inter-examiner differences for paired sextant-level scores were assessed using the Wilcoxon signed-rank test, selected due to the ordinal scale structure and non-normal distribution of the data. For each index and each sextant (or tooth group for sTWI), paired comparisons between Rater 1 and Rater 2 were summarized both numerically and graphically. To improve the clarity of the results presentation, *p*-values were accompanied by explicit indicators of statistical significance, where (S) denotes statistically significant differences (*p* < 0.05) and (NS) denotes non-significant results (*p* ≥ 0.05). Score agreement patterns were additionally explored using confusion matrices and heatmaps to illustrate systematic scoring tendencies.

Sex-stratified trends were explored using linear mixed-effects models fitted separately for each index and anatomical unit (sextant or tooth surface). In these models, tooth-wear score was the dependent variable, sex (female/male) was included as a fixed effect, and random intercepts were specified for patient and examiner to account for repeated measurements within individuals and between raters. Sex-stratified analyses were exploratory and intended only to describe possible distributional patterns. Scores coded as ‘R’ (restored) or missing were excluded. Model assumptions were checked using residual diagnostics; statistical significance was set at *p* < 0.05, without adjustment for multiple testing, reflecting the exploratory nature of the analysis.

Tooth-wear scores are ordinal; however, for interpretability linear mixed-effects models were used, which approximate score differences as continuous [30]. This simplification may slightly affect effect-size estimates and should be considered when interpreting the results.

Given the anonymized nature of the dataset and the lack of key clinical covariates (e.g., age, parafunction, dietary factors), all sex-related and examiner-based comparisons were considered exploratory and unadjusted. *p*-values are reported to describe statistical trends, but results should not be overinterpreted as causal.

## 3. Results

Analysis of the 246 intraoral scans showed that BEWE, sTWI, and TWES 2.0 exhibited distinct scoring patterns when applied to digital models. Although all three indices could be consistently applied to intraoral scans, their score distributions differed in accordance with their diagnostic structures. BEWE produced the narrowest range of values due to its coarse, sextant-based scoring system, whereas sTWI generated greater variability, particularly in anterior teeth and first molars, where surface-level changes are more easily detected. TWES 2.0 demonstrated broader distributions than BEWE, particularly in anterior sextants (due to the additional palatal score) and in some posterior sextants.

Across all indices, estimated marginal means showed relatively consistent internal patterns across sextants. Sextants commonly affected by functional and/or erosive challenges—particularly anterior regions and posterior occlusal surfaces—tended to show higher values in sTWI and TWES 2.0, while BEWE showed less regional differentiation [1,2,28,30].

Exploratory comparisons between sexes revealed only minor and inconsistent variations. Although males showed slightly higher values in selected sextants across indices, these differences were small, lacked a consistent pattern, and were not statistically meaningful. Because age and clinical cofactors were unavailable for anonymized scans, sex-related results remain descriptive and should be interpreted cautiously.

Taken together, these findings suggest that intraoral scans provide a feasible basis for applying BEWE, sTWI, and TWES 2.0, while also demonstrating that the indices emphasize different clinical dimensions of tooth wear [23,39,42,43,44,45,46]. The following sections provide detailed index-specific results supported by tables and figures.

### 3.1. BEWE

**Figure 1:**
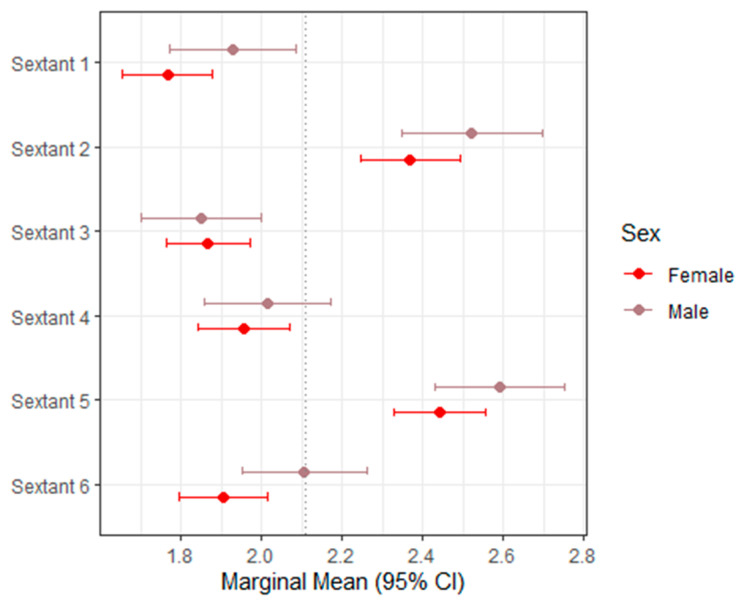
Forest plot of marginal means (95% CI) for BEWE. The dashed vertical line indicates the overall marginal mean.

Table 2 summarizes the mixed-effects model estimates and marginal means for BEWE across sextants, with the corresponding 95% confidence intervals illustrated in Figure 1. BEWE demonstrated a narrow distribution of sextant scores, reflecting the limited resolution of its 0–3 screening scale.

Across the dentition, sex was not a statistically significant predictor for most sextants. The only exception was Sextant 6, where males demonstrated slightly higher scores than females (estimate = +0.201; *p* = 0.038), although the magnitude of this difference was small. In all remaining sextants, the differences between males and females were minimal, inconsistent, and without apparent clinical relevance.

Overall, the pattern indicates that BEWE produces relatively uniform scores across sextants when applied to digital intraoral scans. This supports its feasibility as a digital screening tool, while also underscoring its limited granularity for detecting subtle morphological differences in tooth wear.

### 3.2. sTWI

**Figure 2:**
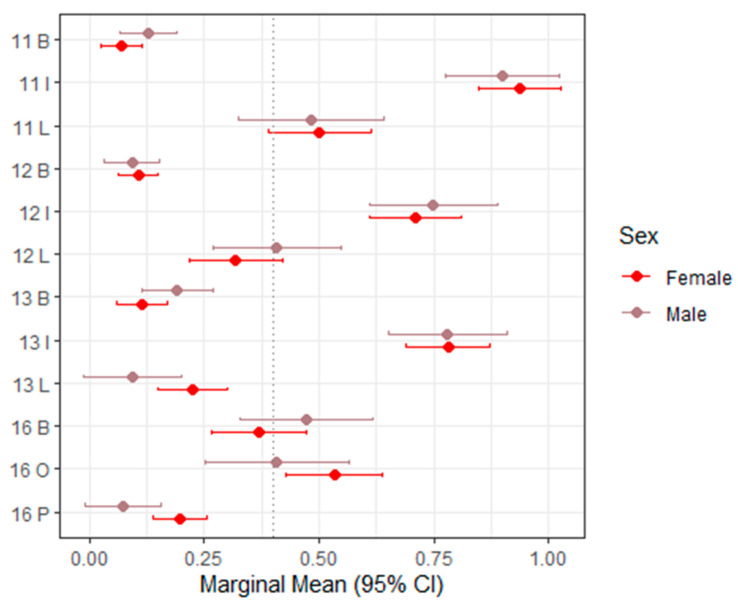
Forest plot of marginal means (95% CI) for sTWI–Upper right (11–16). The dashed vertical line indicates the overall marginal mean. B = buccal, I = incisal, O = occlusal, L = lingual.

**Figure 3:**
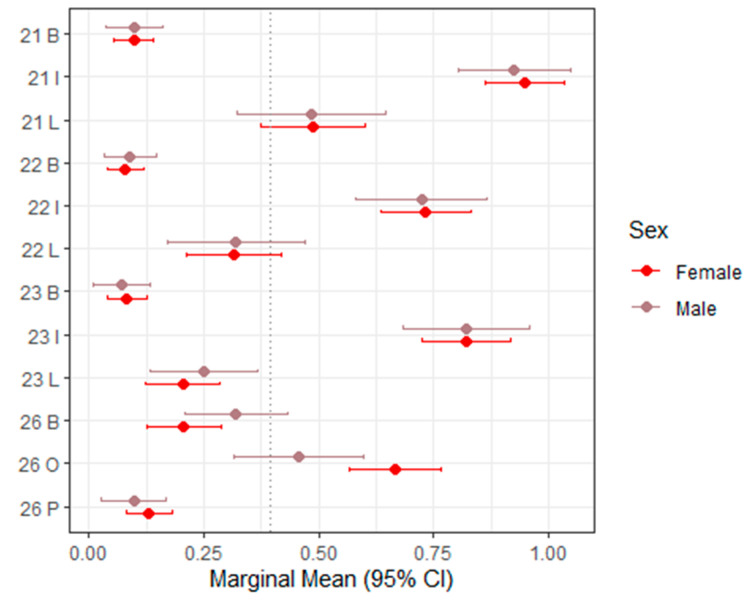
Forest plot of marginal means (95% CI) for sTWI–Upper left (21–26). The dashed vertical line indicates the overall marginal mean. B = buccal, I = incisal, O = occlusal, L = lingual.

**Figure 4:**
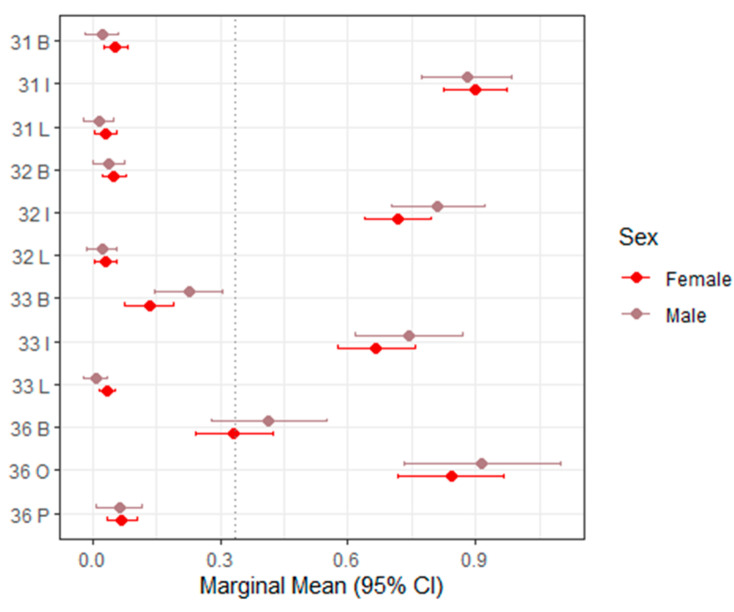
Forest plot of marginal means (95% CI) for sTWI–Lower left (31–36). The dashed vertical line indicates the overall marginal mean. B = buccal, I = incisal, O = occlusal, L = lingual.

**Figure 5:**
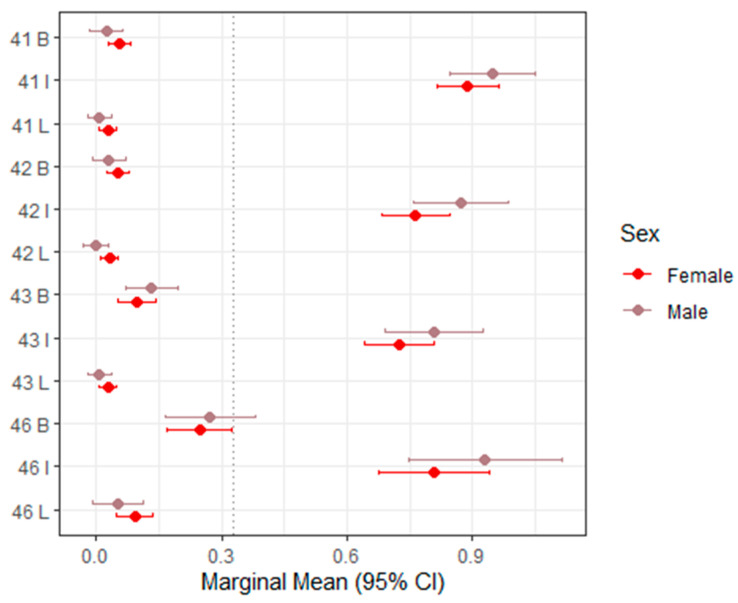
Forest plot of marginal means (95% CI) for sTWI–Lower right (41–46). The dashed vertical line indicates the overall marginal mean. B = buccal, I = incisal, L = lingual.

Table 3 presents the mixed-effects model estimates and marginal means for sTWI across all evaluated tooth surfaces, with corresponding 95% confidence intervals visualized in Figure 2 (upper right, teeth 11–16), Figure 3 (upper left, teeth 21–26), Figure 4 (lower left, teeth 31–36), and Figure 5 (lower right, teeth 41–46).

Overall, sTWI values showed moderate variability across tooth surfaces, reflecting the greater scoring granularity of this index compared with BEWE. In most regions, sex was not a statistically significant predictor of sTWI scores. The majority of *p*-values exceeded 0.05, and the estimated differences between males and females were small or close to zero, indicating minimal systematic variation.

A limited number of isolated sites demonstrated statistically significant sex-related differences. For example, at the palatal surface of tooth 16 and the occlusal surface of tooth 26, males exhibited significantly lower scores than females (estimate = –0.123; *p* = 0.0166 [S]). These findings, however, did not follow a broader anatomical or functional pattern.

Across most evaluated tooth positions, the direction of sex differences was inconsistent. In some locations (e.g., 16 buccal, 13 buccal, 26 buccal, and 36 buccal), males showed slightly higher marginal means. In contrast, at other sites (e.g., 16 occlusal, 13 lingual, and 26 occlusal), females demonstrated slightly higher scores.

Taken together, these variations appear random rather than systematic. The overall pattern indicates that sex has minimal and clinically negligible influence on sTWI-based tooth wear estimates when assessed from intraoral scans. The index maintained a relatively stable distribution across tooth regions, supporting its suitability for digital scoring while highlighting its limited discriminatory power in identifying sex-associated differences.

### 3.3. TWES 2.0

**Figure 6:**
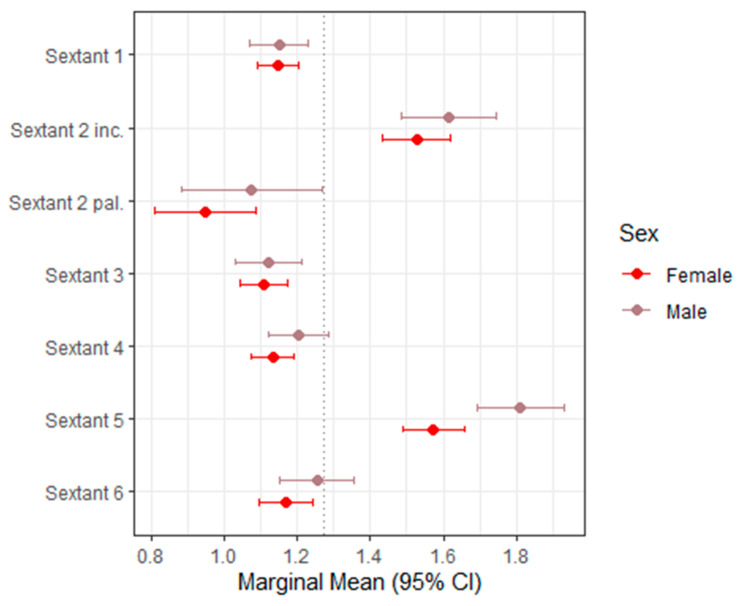
Forest plot of marginal means (95% CI) for TWES 2.0. The dashed vertical line indicates the overall marginal mean.

Table 4 presents the mixed-effects model estimates and marginal means for TWES 2.0 across sextants, with the corresponding 95% confidence intervals displayed in Figure 6 as a forest plot.

Overall, TWES 2.0 showed a slightly wider scoring range than BEWE, reflecting its greater sensitivity to moderate and severe morphological changes. Despite this broader distribution, sex was not a significant predictor of TWES scores in most sextants. For the majority of regions, estimated differences between males and females were small, non-significant, and lacked directional consistency.

A statistically significant sex effect was observed only in Sextant 5, where males demonstrated higher TWES scores than females (estimate = +0.237; *p* = 0.0016 [S]). The marginal mean for males in this sextant was 1.81, compared with 1.57 for females. Although statistically significant, this difference corresponds to less than one TWES category and may not represent a clinically meaningful discrepancy.

In all other sextants (1, 2 incisal, 2 palatal, 3, 4, and 6), sex differences were not statistically significant (all *p* ≥ 0.05 [NS]). Estimated marginal means showed only minimal variation between males and females, and confidence intervals overlapped substantially, indicating no systematic pattern of increased or decreased wear associated with sex.

Taken together, these findings suggest that sex has a limited and inconsistent influence on TWES 2.0 scores when evaluated on digital intraoral scans. TWES 2.0 demonstrated relatively consistent score patterns across sextants, reinforcing its applicability for digital screening while also confirming that sex-based differences—where present—are small and isolated rather than global or diagnostic.

## 4. Discussion

### 4.1. Summary of Main Findings

This study compared three commonly used tooth-wear indices—BEWE, sTWI, and TWES 2.0—applied to digital intraoral scans, and showed that they differ markedly in their scoring behavior, sensitivity, and regional emphasis. These results are consistent with previous reports indicating that tooth-wear indices are not interchangeable and may yield different outcomes depending on their scoring resolution, anatomical focus, and sensitivity to specific wear mechanisms [15,23,26,28,30,33,42,43,44,45,46,47].

Although formal inter-rater reliability metrics such as the Intraclass Correlation Coefficient (ICC) were not included, inter-examiner agreement was explored using paired comparisons, confusion matrices, and heatmaps to assess scoring patterns and variability. BEWE and TWES 2.0 generally produced more consistent results between examiners, whereas sTWI showed greater variability, particularly in surface-specific assessments [17,26,29,32,35,47].

The study also included exploratory sex-stratified comparisons. However, the absence of age and other clinical cofactors in this retrospective dataset prevents meaningful interpretation of biological sex differences. Observed differences must be regarded as descriptive only.

### 4.2. Comparison with Previous Studies

BEWE, owing to its coarse 0–3 scoring scale, produced the narrowest score distribution [16,17,23]. This is consistent with earlier reports indicating that BEWE is useful for rapid clinical screening but lacks granularity for fine-scale differentiation [15,26,32].

By contrast, sTWI produced greater variability, consistent with characteristics of the original Tooth Wear Index [18,28,33,34]. Previous studies have reported that TWI-based indices detect localized enamel and dentin loss effectively but exhibit higher examiner variability [30,33], particularly on surfaces where fine morphological distinctions are required.

TWES 2.0 demonstrated an intermediate performance [15,20,29,38,39,40,41]. It produced a broader score distribution than BEWE but was more consistent than sTWI, aligning with published data showing that TWES 2.0 was designed to strengthen diagnostic clarity while maintaining usability. 

Regarding sex, studies have reported mixed results [2,4,8,9,48]. Some found greater wear among males [2,4,8], while others found no significant sex differences when adjusting for age and lifestyle factors [9,32,48]. Without age data, our study cannot contribute to this debate.

### 4.3. Methodological Considerations

It is important to emphasize that, despite the use of digital intraoral scans, the present study relied on examiner-based visual scoring rather than automated or quantitative digital measurement techniques.

#### 4.3.1. Influence of Examiner Calibration and Scoring Structure

Indices with broader scoring categories, such as BEWE and TWES 2.0, tend to show more consistent scoring patterns, because they rely on coarse ordinal distinctions and sextant-level evaluation [16,29]. Surface-specific systems such as sTWI, however, introduce greater variability, particularly when applied to digital meshes where enamel translucency, surface gloss, and color cues are reduced or absent [33].

These patterns have also been confirmed in digital model validation studies showing that finer index granularity increases examiner disagreement [11,23,28].

#### 4.3.2. Strengths of Digital Intraoral Scans

Digital intraoral scans offer notable diagnostic advantages [11,13,14,49]. High-resolution 3D meshes allow unrestricted rotation, zooming, and magnification beyond what is possible clinically, improving the visibility of posterior occlusal morphology and palatal surfaces [13,49]. The ability to stabilize the image, adjust angles, and compare contralateral teeth enhances consistency and reduces lighting and positioning bias [11]. Digital models also permit retrospective scoring and application of multiple indices without repeat patient visits [14].

#### 4.3.3. Diagnostic Limitations of Digital Scans

Despite their strengths, digital models present known limitations [12,50]. STL meshes lack optical information such as translucency and gloss, and even colorized PLY files may not accurately reflect natural enamel appearance [50]. Early erosive texture changes and enamel softening often remain undetectable [12]. Reflective artifacts and stitching errors may distort occlusal anatomy [11,13].

The 2017 European Consensus Statement emphasized that wear indices were developed for clinical evaluation and should not be used to quantify disease progression solely on the basis of digital scans [21].

### 4.4. Clinical and Research Implications

The findings of this study demonstrate that the choice of index has a direct and substantial influence on the interpretation of digital tooth-wear patterns [15,26,28,30,33]. The three indices assessed here are not interchangeable; each captures different dimensions of wear and therefore leads to distinct diagnostic impressions. Broad-scale indices such as BEWE and TWES 2.0 appear to offer more consistent scoring patterns in digital workflows because their coarse categorical structure reduces examiner-dependent variation. In contrast, fine-scale indices like sTWI remain highly sensitive to examiner interpretation and are affected by the inherent limitations of digital mesh models, including reduced optical surface information [15]. Importantly, sex-related differences cannot be meaningfully evaluated in digital datasets that lack age information, as tooth wear is strongly age-dependent and confounded by numerous behavioral and clinical variables. For clinical applications, BEWE appears most appropriate for rapid digital screening, whereas TWES 2.0 provides a more structured framework for cases requiring targeted assessment of severity and location. sTWI, due to its surface-level granularity, should be reserved for settings in which detailed clinical context and tactile examination can supplement digital evaluation [15].

### 4.5. Limitations

The interpretation of results is constrained by the retrospective nature of the dataset and the lack of key variables. Tooth wear is multifactorial, involving erosive, attritional, abrasive, and parafunctional components [5,6,7]. Behavioral and biological factors known to affect wear—bruxism [9,51,52], intrinsic acid exposure [53,54], salivary buffering [55,56], dietary acids [4,56], and occlusal load patterns [8]—were not available.

Their absence makes etiological interpretation impossible and limits conclusions to descriptive observations.

Scanner type (Medit i500 vs. iTero Element 2) was not included as a covariate. Although both systems generate high-resolution full-arch meshes suitable for tooth-wear assessment, subtle device-related differences in mesh quality cannot be excluded as a source of additional variability. Future studies could explore whether scanner type influences scoring behavior, as different devices may produce meshes with slightly different surface characteristics.

### 4.6. Future Directions

Future research should focus on age-matched cohorts and incorporate detailed behavioral, functional, and clinical cofactors to allow more accurate interpretation of tooth-wear patterns and reduce confounding. Prospective longitudinal studies using repeated digital scans are needed to quantify progression rather than rely on cross-sectional comparisons. Integrating automated, machine-learning–based wear detection systems may also enhance diagnostic objectivity and reduce examiner-related variability, particularly for fine-scale indices [23,42,43,44,45,46]. Continued improvements in scanner resolution, color accuracy, and surface-texture mapping will likely increase the diagnostic reliability of digital tooth-wear assessment, bringing digital workflows closer to fully replacing certain aspects of traditional clinical evaluation.

## 5. Conclusions

Within the limitations of this retrospective dataset, BEWE, sTWI, and TWES 2.0 demonstrated distinct scoring patterns when applied to intraoral scans, suggesting that these indices are not interchangeable and capture different dimensions of tooth wear. BEWE and TWES 2.0 demonstrated more consistent scoring behavior between examiners, supporting their suitability for digital screening and standardized clinical use. In contrast, sTWI provided greater scoring variability, likely reflecting its finer surface-level resolution and increased examiner dependency.

Sex-related differences were small, inconsistent across indices, and should not be overinterpreted, particularly given the lack of age data and absence of relevant clinical cofactors. Therefore, this dataset does not allow reliable conclusions about true sex-related patterns of tooth wear.

Overall, these findings highlight the importance of selecting the appropriate index according to study aims and clinical context. Future research should include age-matched samples and integrate dietary, behavioral, and functional variables to better understand the multifactorial contributors to tooth wear and to validate the performance of digital assessment methods in longitudinal settings.

## Figures and Tables

**Table 1:** Baseline characteristics—sex.

Variable	Parameter	Overall (N = 246)
Sex	Female	66.2% (N = 129)
	Male	33.8% (N = 66)
	Unknown	20.7% (N = 51)

**Table 2:** Mixed models and marginal means (BEWE).

Variable	Estimate (95% CI)	Marginal Mean Female (95% CI)	Marginal Mean Male (95% CI)	*p*-Value
Sextant 1	0.162 (−0.030–0.355)	1.77 (1.66–1.88)	1.93 (1.77–2.09)	0.098
Sextant 2	0.155 (−0.058–0.367)	2.37 (2.24–2.49)	2.52 (2.35–2.70)	0.1535
Sextant 3	−0.019 (−0.201–0.163)	1.87 (1.76–1.97)	1.85 (1.70–2.00)	0.8389
Sextant 4	0.057 (−0.137–0.251)	1.96 (1.84–2.07)	2.01 (1.86–2.17)	0.5602
Sextant 5	0.149 (−0.048–0.346)	2.44 (2.33–2.56)	2.59 (2.43–2.75)	0.1366
Sextant 6	0.201 (0.011–0.391)	1.91 (1.80–2.02)	2.11 (1.95–2.26)	0.0381 *
Total BEWE	0.609 (−0.256–1.474)	12.19 (11.69–12.70)	12.80 (12.10–13.51)	0.1664

(*) = Statistically significant (*p* < 0.05).

**Table 3:** Mixed models and marginal means (sTWI). B—buccal surface, O—occlusal surface, P—palatinal, I—incisal surface, L—lingual surface, and V—vestibular surface.

Variable	Estimate (95% CI)	Marginal Mean Female (95% CI)	Marginal Mean Male (95% CI)	*p*-Value
16 B	0.102 (−0.075–0.279)	0.37 (0.27–0.47)	0.47 (0.33–0.62)	0.2555
16 O	−0.126 (−0.314–0.063)	0.53 (0.43–0.64)	0.41 (0.25–0.56)	0.1893
16 P	−0.123 (−0.224–−0.023)	0.20 (0.14–0.26)	0.07 (−0.01–0.16)	0.0166 *
13 B	0.076 (−0.019–0.171)	0.12 (0.06–0.17)	0.19 (0.11–0.27)	0.1163
13 I	−0.001 (−0.160–0.157)	0.78 (0.69–0.87)	0.78 (0.65–0.91)	0.9873
13 L	−0.131 (−0.260–−0.002)	0.23 (0.15–0.30)	0.09 (−0.01–0.20)	0.0472 *
12 B	−0.014 (−0.089–0.061)	0.11 (0.06–0.15)	0.09 (0.03–0.15)	0.7162
12 I	0.039 (−0.132–0.210)	0.71 (0.61–0.81)	0.75 (0.61–0.89)	0.6516
12 L	0.090 (−0.082–0.261)	0.32 (0.22–0.42)	0.41 (0.27–0.55)	0.3051
11 B	0.057 (−0.020–0.133)	0.07 (0.03–0.12)	0.13 (0.07–0.19)	0.1442
11 I	−0.039 (−0.191–0.114)	0.94 (0.85–1.03)	0.90 (0.77–1.02)	0.6189
11 L	−0.019 (−0.212–0.175)	0.50 (0.39–0.61)	0.48 (0.33–0.64)	0.847
21 B	0.001 (−0.075–0.076)	0.10 (0.05–0.14)	0.10 (0.04–0.16)	0.9888
21 I	−0.023 (−0.172–0.125)	0.95 (0.86–1.03)	0.93 (0.80–1.05)	0.757
21 L	−0.003 (−0.201–0.194)	0.49 (0.37–0.60)	0.48 (0.32–0.65)	0.9727
22 B	0.010 (−0.059–0.079)	0.08 (0.04–0.12)	0.09 (0.03–0.15)	0.7653
22 I	−0.009 (−0.183–0.165)	0.73 (0.63–0.83)	0.72 (0.58–0.87)	0.9186
22 L	0.003 (−0.178–0.184)	0.32 (0.21–0.42)	0.32 (0.17–0.47)	0.9737
23 B	−0.010 (−0.085–0.064)	0.08 (0.04–0.13)	0.07 (0.01–0.13)	0.7853
23 I	−0.000 (−0.169–0.168)	0.82 (0.73–0.92)	0.82 (0.68–0.96)	0.9994
23 L	0.045 (−0.096–0.187)	0.20 (0.12–0.29)	0.25 (0.13–0.37)	0.5285
26 B	0.114 (−0.024–0.252)	0.21 (0.13–0.29)	0.32 (0.21–0.43)	0.1059
26 O	−0.210 (−0.384–−0.037)	0.67 (0.57–0.77)	0.46 (0.31–0.60)	0.0175 *
26 P	−0.032 (−0.118–0.055)	0.13 (0.08–0.18)	0.10 (0.03–0.17)	0.4677
36 B	0.081 (−0.082–0.244)	0.33 (0.24–0.42)	0.41 (0.28–0.55)	0.3302
36 O	0.072 (−0.150–0.293)	0.84 (0.72–0.97)	0.91 (0.73–1.10)	0.5217
36 P	−0.005 (−0.070–0.059)	0.07 (0.03–0.11)	0.06 (0.01–0.12)	0.8692
33 B	0.094 (−0.004–0.192)	0.13 (0.08–0.19)	0.23 (0.15–0.31)	0.0607
33 I	0.076 (−0.080–0.232)	0.67 (0.58–0.76)	0.74 (0.62–0.87)	0.3397
33 L	−0.028 (−0.060–0.005)	0.04 (0.02–0.05)	0.01 (−0.02–0.03)	0.1004
32 B	−0.013 (−0.059–0.034)	0.05 (0.02–0.08)	0.04 (0.00–0.08)	0.5915*
32 I	0.094 (−0.041–0.228)	0.72 (0.64–0.80)	0.81 (0.70–0.92)	0.1723
32 L	−0.008 (−0.052–0.035)	0.03 (0.01–0.06)	0.02 (−0.01–0.06)	0.7075
31 B	−0.031 (−0.079–0.016)	0.05 (0.03–0.08)	0.02 (−0.02–0.06)	0.1932
31 I	−0.020 (−0.149–0.108)	0.90 (0.82–0.97)	0.88 (0.77–0.98)	0.7539
31 L	−0.016 (−0.058–0.026)	0.03 (0.01–0.06)	0.02 (−0.02–0.05)	0.4597
41 B	−0.031 (−0.079–0.016)	0.05 (0.03–0.08)	0.02 (−0.02–0.06)	0.1932
41 I	0.059 (−0.067–0.186)	0.89 (0.81–0.96)	0.95 (0.84–1.05)	0.3559
41 L	−0.020 (−0.053–0.014)	0.03 (0.01–0.05)	0.01 (−0.02–0.03)	0.2476
42 B	−0.020 (−0.068–0.027)	0.05 (0.02–0.08)	0.03 (−0.01–0.07)	0.403
42 I	0.110 (−0.029–0.250)	0.76 (0.68–0.84)	0.87 (0.76–0.99)	0.1205
42 L	−0.031 (−0.068–0.005)	0.03 (0.01–0.05)	−0.00 (−0.03–0.03)	0.0944
43 B	0.035 (−0.041–0.110)	0.10 (0.05–0.14)	0.13 (0.07–0.19)	0.3665
43 I	0.081 (−0.063–0.225)	0.73 (0.64–0.81)	0.81 (0.69–0.92)	0.2684
43 L	−0.019 (−0.053–0.014)	0.03 (0.01–0.05)	0.01 (−0.02–0.04)	0.2553
46 B	0.025 (−0.106–0.157)	0.25 (0.17–0.32)	0.27 (0.17–0.38)	0.7033
46 O	0.123 (−0.102–0.348)	0.81 (0.68–0.94)	0.93 (0.75–1.11)	0.282
46 L	−0.040 (−0.114–0.035)	0.09 (0.05–0.13)	0.05 (−0.01–0.11)	0.2924

(*) = Statistically significant (*p* < 0.05).

**Table 4:** Mixed models and marginal means (TWES 2.0).

Variable	Estimate (95% CI)	Marginal Mean Female (95% CI)	Marginal Mean Male (95% CI)	*p*-Value
Sextant 1	0.003 (−0.097–0.102)	1.15 (1.09–1.21)	1.15 (1.07–1.23)	0.9559
Sextant 2 inc.	0.087 (−0.074–0.247)	1.53 (1.43–1.62)	1.61 (1.48–1.74)	0.288
Sextant 2 pal.	0.125 (−0.111–0.362)	0.95 (0.81–1.09)	1.08 (0.88–1.27)	0.2969
Sextant 3	0.013 (−0.098–0.124)	1.11 (1.04–1.17)	1.12 (1.03–1.21)	0.8175
Sextant 4	0.072 (−0.029–0.172)	1.13 (1.07–1.19)	1.20 (1.12–1.29)	0.161
Sextant 5	0.237 (0.091–0.383)	1.57 (1.49–1.66)	1.81 (1.69–1.93)	0.0016 *
Sextant 6	0.085 (−0.039–0.209)	1.17 (1.10–1.24)	1.25 (1.15–1.35)	0.1801

(*) = Statistically significant (*p* < 0.05).

## Data Availability

All the data generated and analyzed during this study are included in this published article. The original intraoral scans are not publicly available because of ethical and privacy restrictions but are available from the corresponding author upon reasonable request.

## Abbreviations

BEWE	Basic Erosive Wear Examination
sTWI	simplified Tooth Wear Index
TWES 2.0	Tooth Wear Evaluation System 2.0
